# Use of ^11^C-Choline positron emission tomography/computed tomography to investigate the mechanism of choline metabolism in lung cancer

**DOI:** 10.3892/mmr.2015.3200

**Published:** 2015-01-14

**Authors:** ZHAOQIN HUANG, JUN RUI, XIN LI, XIANGJIAO MENG, QINGWEI LIU

**Affiliations:** 1 Department of Radiology, Provincial Hospital Affiliated to Shandong University, Jinan, Shandong 250021, P.R. China; 2 Department of Radiology, People’s Liberation Army 520 Hospital, Mianyang, Sichuan 621000, P.R. China; 3 Department of Radiation Oncology, Shandong Cancer Hospital and Institute, Jinan, Shandong 250117, P.R. China

**Keywords:** lung cancer, choline acetyltransferase, choline kinase, ^11^C-Choline, positron emission tomography

## Abstract

The present study was conducted to investigate the ^11^C-Choline metabolic mechanism and examine the association between ^11^C-Choline metabolism and uptake in different pathological types of lung cancer. A total of 18 tumor specimens and corresponding normal lung tissues were collected from patients who were diagnosed with lung cancer using ^11^C-Choline positron emission tomography (PET)/computed tomography (CT) imaging between January 2007 and December 2008 at the Medical Imaging Center of the Provincial Hospital Affiliated to Shandong University. The diagnosis was further confirmed pathologically following surgery. Reverse transcription polymerase chain reaction and western blotting were used to investigate the expression of choline acetyltransferase (ChAT) and choline kinase α (ChoK) in lung cancer tissue and normal lung tissue. The ^11^C-Choline PET/CT data were analyzed visually and semiquantitatively. Compared with the expression in the normal lung tissues, the mRNA and protein expression of ChAT and ChoK increased in nine and 14 of the 18 lung tumors, respectively. A total of eight of the 18 tumors exhibited significantly increased expression, while three exhibited no expression of ChoK and ChAT. All lung cancer lesions were visualized with ^11^C-Choline PET/CT imaging. The phosphorylation and acetylation pathways of choline metabolism may be important in ^11^C-Choline uptake and metabolism in different pathological types of lung cancer.

## Introduction

Lung cancer is one of the most common types of cancer and was the leading cause of cancer-associated mortality in males and the second in females in 2008 (1). However, the 5-year survival rate of lung cancer is up to 53% if the disease remains localized at diagnosis. However, only 15% of cases of lung cancer are diagnosed at an early stage (1). Since the management of lung cancer is dependent on the pathological type of the tumor and stages, it is critical to make a precise diagnosis and staging when the disease is first diagnosed.

Positron emission tomography with (^18^F)fluorodeoxyglucose (^18^F-FDG PET) has been established as an accurate non-invasive method in staging and a useful tool in the management of lung cancer (2–4). However, ^18^F-FDG PET has been revealed as having a low sensitivity and/or specificity for the assessment of certain types of cancer, thus there is a requirement to develop new tracers for PET imaging (5). Previously, ^11^C-labeled choline (^11^C-Choline) has been described as a promising PET tracer for tumor detection and staging as well as for the monitoring of therapeutic response in certain types of carcinoma (6–8).

Choline is a quaternary ammonium base, which is a precursor of cell membrane phospholipids. Choline is metabolized *in vivo* through three pathways, including phosphorylation, acetylation and oxidation (9). Choline kinase α (ChoK) catalyzes choline phosphorylation through ATP, producing phosphorylcholine. Phosphorylcholine is an intracellular storage pool of choline and it is further incorporated into phosphatidylcholine, lecithin, a major phospholipid of all membranes. Choline acetyltransferase (ChAT) catalyzes the reaction of acetyl coenzyme A with choline to produce acetylcholine. Choline is also oxidized to betaine aldehyde, which is further converted into betaine by the enzyme system of choline oxidase (choline dehydrogenase and betaine aldehyde dehydrogenase), predominantly in the liver and kidneys. The majority of studies suggest that phosphorylation is the only pathway of ^11^C-Choline metabolism in cancer cells (10,11), however, Song *et al* (12,13) have demonstrated that acetylation is another pathway of choline metabolism in small cell lung cancer and acetylcholine acts as an autocrine growth factor in small cell lung cancer. Nevertheless, the metabolism of choline in lung cancer cells requires further elucidation. The present study was designed to determine the expression of ChAT and ChoK in order to clarify the roles of phosphorylation and acetylation pathways in ^11^C-Choline metabolism in different types of lung cancer.

## Materials and methods

### Patients

A total of 18 patients (14 male and four female; mean age, 56.9 years old; age range, 23–72 years) diagnosed with lung cancer at the Medical Imaging Center of the Provincial Hospital Affiliated to Shandong University (Shandong, China) between January 2007 and December 2008 were included in the present study. The patients with suspected lung cancer underwent ^11^C-Choline PET and computed tomography (PET/CT) examination. Surgical resection was performed within 1 week. All patients were confirmed to have lung cancer based on pathology. Lung cancer tissues and the corresponding normal lung tissues (peripheral areas of the surgically removed tissues) were sampled in all patients for examination of choline metabolism. The present study was approved by the Shandong Provincial Hospital Institutional Review Board (Jinan, China) and all patients signed informed consent.

### Reverse transcription polymerase chain reaction (RT-PCR)

RT-PCR was used to determine the mRNA expression of ChAT and ChoK in the lung tumor and normal tissues. Total RNA was extracted from the tissues with TRIzol reagent (Millennium Biomedical, Inc., Pomona, CA, USA) according to the manufacturer’s instructions. The PCR primers for ChAT, ChoK and β-actin amplification are shown in Table 1. The PCR conditions were denaturation at 94°C for 2 min, followed by 28 cycles at 94°C for 30 sec, 58°C for 30 sec and 72°C for 40 sec. β-actin mRNA was used to normalize the expression of ChAT and ChoK mRNA.

### SDS-polyacrylamide gel electrophoresis (PAGE) and western blot analysis

Western blot analysis was used to measure the protein expression of ChAT and ChoK in the lung tumor and normal tissues. The tissues were rinsed with cold phosphate-buffered saline (Beijing Zhongshan Golden Bridge Biotechnology Co., Ltd., Beijing, China) and homogenized and lysed with cell lysis buffer (50 mmol/l Tris HCl, pH 8.0, 150 mmol/l NaCl, 0.1% SDS, 100 μg/ml phenylmethylsulfonyl fluoride, 1 μg/ml aprotinin and 1% NP-40). The tissue extracts were quantified using the bicinchoninic method and 30 μg of tissue extracts were loaded onto 10% SDS-PAGE gels. Following electrophoresis, proteins were transferred onto nitrocellulose membranes (Bio-Rad, Hercules, CA, USA). Following being blocked and washed, the membranes were treated with mouse monoclonal antibody against human ChAT (MAB5350; EMD Millipore, Billerica, MA, USA) with a 1:5,000 dilution or rabbit polyclonal antibody against human ChoK (sc-32907; Santa Cruz Biotechnology, Inc., Dallas, TX, USA) with a 1:2,000 dilution at 4°C for 12 h, followed by incubation with peroxidase-labeled goat anti-rabbit immunoglobulin G (474-1516; KPL, Gaithersburg, MA, USA) with a 1:2,000 dilution for 1 h at room temperature. The immunoreactive bands were visualized with enhanced chemiluminescence (Santa Cruz Biotechnology, Inc.). β-actin (Santa Cruz Biotechnology, Inc.) was used to normalize the quantity of proteins on the blot.

### ^11^C-Choline PET/CT imaging

^11^C-CO_2_ was produced with a MINItrace cyclotron (GE Healthcare, Piscataway, NJ, USA). ^11^C-Choline was synthesized using the solid-phase method as described by Pascali *et al* (14) in a modified commercial synthesis module (TRACERlab FXc; GE Healthcare). The radiochemical purity of the ^11^C-Choline was evaluated to be >95% with a high-performance liquid chromatography radiodetector (TRACERlab FXc; GE Healthcare).

All PET scans were obtained using a PET/CT scanner (Discovery LS; GE Healthcare). Each patient was injected with 7.4 MBq/kg of ^11^C-Choline intravenously 5 min prior to imaging. PET images were captured in the supine position over two bed positions (3 min per position) from the upper neck to the lower edge of the liver, or six bed positions (whole body) when additional imaging revealed distant metastasis. The parameters of the multidetector helical CT scan were 140 kV, 80 mA, 0.8 sec per tube rotation, 5 mm slice thickness, 6:1 pitch and 11.25 mm/sec table speed. PET images were reconstructed with the iterative reconstruction ordered-subset expectation maximization likelihood algorithm of the manufacturer following attenuation correction based on the CT dataset. Consecutive transverse PET/CT slices at 4.25 mm thickness were generated.

### Image analysis

All PET images were analyzed with the dedicated software (Xeleris, version 1.1363; GE Healthcare) that allows review of PET, CT and fused-image data. PET images were initially assessed visually using the transaxial, sagittal and coronal displays by two experienced nuclear medicine physicians who were blinded to the clinical data and the results of the previous imaging studies. The circular region of interest (ROI) was drawn over the abnormal areas with increased ^11^C-Choline uptake in the lung tumor and the standardized uptake values (SUVmax and SUVmean) were measured. At least three circular (1 cm in diameter) ROI were also drawn in the normal lung tissues at the slice same to the tumor and the highest SUVmax was accepted as the SUVmax of the normal lung tissue. The radioactivities in the lung tumor (T) and normal tissue (NT) were measured and the T/NT ratios were calculated. The PET/CT findings were compared with the pathological results.

### Statistical analysis

Statistical analysis was performed using Statistical Package for the Social Sciences software (SPSS for Windows 13.0; SPSS, Inc., Chicago, IL, USA). The data of SUVmean, SUVmax and T/NT are expressed as the mean ± standard deviation and the two-sample t-test was used for comparison between tumors and normal tissues. P<0.05 was considered to indicate a statistically significant difference.

## Results

### Pathological findings

Lung cancer was confirmed pathologically in all 18 patients, including eight squamous cell carcinomas, six adenocarcinomas, two atypical carcinoids, one small cell carcinoma and one mucoepidermoid carcinoma.

### ChAT and ChoK mRNA expression in lung cancer

**Figure 1:**
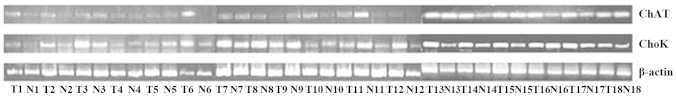
ChAT and ChoK mRNA expression in lung lesion biopsies. RT-PCR was performed to detect ChAT and ChoK mRNA expression on total RNA prepared from the lung lesion biopsies (T) and normal lung tissues (N). Primers used for RT-PCR are described in Table 1. β-actin mRNA was amplified and used for standardization. ChaT, choline acetyltransferase; ChoK choline kinase α; RT-PCR, reverse transcription polymerase chain reaction.

RT-PCR was performed to determine the ChAT and ChoK mRNA expression in the tumor and normal tissues. The primers used for RT-PCR are shown in Table 1. As shown in Fig. 1 and compared with the expression in normal lung tissues, the mRNA expression of ChAT increased in nine out of the 18 lung tumors, including four adenocarcinomas, two squamous cell carcinomas, one atypical carcinoid, one small cell carcinoma and one mucoepidermoid carcinoma (T1, T2, T6, T8, T11, T12, T14, T16 and T17; Fig. 1). Increased mRNA expression of ChoK was found in 14 out of the 18 lung tumors, including five adenocarcinomas, seven squamous cell carcinomas, one atypical carcinoid and one small cell carcinoma (T1, T2, T3, T6, T7, T8, T9, T11, T12, T13, T14, T15, T17 and T18; Fig. 1).

### ChAT and ChoK protein expression in lung cancer

**Figure 2:**
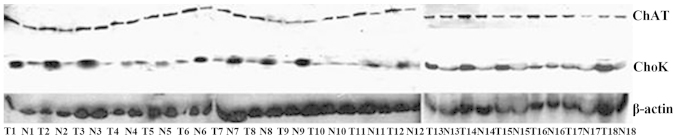
ChAT and ChoK protein expression in lung tumor biopsies. Western blot analysis was performed to detect ChAT and ChoK protein expression in lung tumor biopsies (T) and normal lung tissues (N). Mouse antibody against human ChAT was diluted 1:5,000 and rabbit antibody against human ChoK was diluted 1:2,000. β-actin protein was used for standardization. ChaT, choline acetyltransferase; ChoK choline kinase α.

Western blot analysis was performed to detect the ChAT and ChoK protein expression. As compared with the expression in the normal lung tissues, the ChAT protein expression was upregulated in nine out of the 18 lung tumors (T1, T2, T6, T8, T11, T12, T14, T16 and T17; Fig. 2) and the ChoK protein expression increased in 14 out of the 18 lung tumors (T1, T2, T3, T6, T7, T8, T9, T11, T12, T13, T14, T15, T17 and T18; Fig. 2).

**Figure 3:**
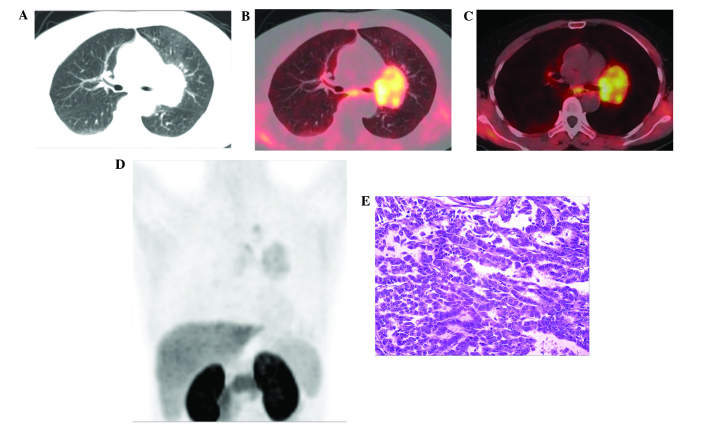
Atypical carcinoid of primary lung cancer in 57-year-old female without expression of ChoK and ChAT. (A) Computed tomography transverse lung window imaging reveals the mass of left lung hilum with bronchial obstruction. (B–D) Positron emission tomography/computed tomography imaging demonstrates ^11^C-Choline uptake with maximum standardized uptake value of 5.4 within mass. (E) Hematoxylin and eosin staining graph reveals that the tumor is an atypical carcinoid (magnification, ×400). ChaT, choline acetyltransferase; ChoK choline kinase α.

**Figure 4:**
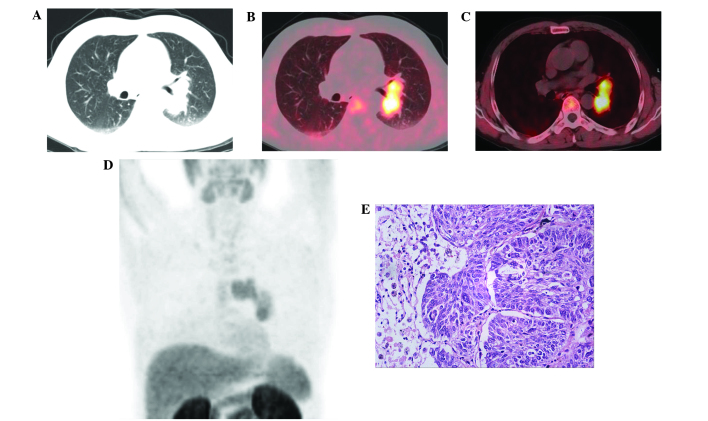
Squamous cell carcinoma of primary lung cancer in a 47-year-old male with high expression of ChoK and ChAT. (A) Computed tomography transverse lung window imaging reveals the mass of left lung hilum with bronchial obstruction. (B–D) Positron emission tomography/computed tomography imaging demonstrates ^11^C-Choline uptake with maximum standardized uptake value of 5.51 within mass. (E) Hematoxylin and eosin staining graph reveals that the tumor is a squamous cell carcinoma (magnification, ×400). ChaT, choline acetyltransferase; ChoK choline kinase α.

**Figure 5:**
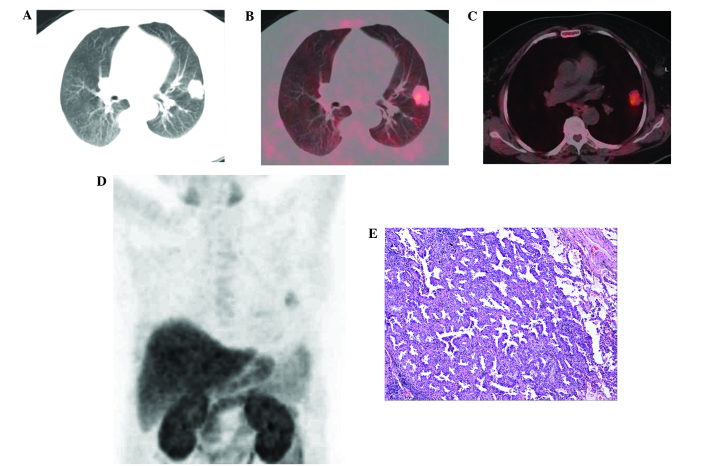
Adenocarcinoma of primary lung cancer in a 59-year-old male with high expression of ChoK and ChAT. (A) Computed tomography transverse lung window imaging reveals the nodule of the superior lobe of left lung. (B–D) Positron emission tomography/computed tomography imaging demonstrates ^11^C-Choline uptake with maximum standardized uptake value of 2.81 within nodule. (E) Hematoxylin and eosin staining graph reveals that the tumor is an adenocarcinoma (magnification, ×400). ChaT, choline acetyltransferase; ChoK choline kinase α.

A total of eight of the 18 lung tumors exhibited a significant increase in the mRNA and protein expression of ChoK and ChAT, including four adenocarcinomas, two squamous cell carcinomas, one atypical carcinoid and one small cell carcinoma. A total of three of the 18 lung tumors (one squamous cell carcinoma, one adenocarcinoma and one atypical carcinoid) did not exhibit expression of ChAT and ChoK, which were assigned to group 1 (Fig. 3). The other 15 lung tumors with high expression of ChoK and/or ChAT were assigned to group 2 (Figs. 4 and 5).

### Characteristics and semiquantitative analysis of ^11^C-Choline PET/CT imaging

All lung cancer lesions were visualized using the ^11^C-Choline PET/CT imaging (Figs. 3, 4 and 5). PET/CT also revealed metastases in the lung hilar lymph nodes (13/17) and mediastinal lymph nodes (10/13) with a clear focal accumulation of ^11^C-Choline.

Quantification revealed that the values of SUVmean, SUVmax and T/NT were 3.55±1.63, 4.24±1.92 and 5.78±1.85, respectively, for all the lung tumors (data not shown). For the eight squamous cell carcinomas, the SUVmean, SUVmax and T/NT values were 3.61±1.90, 4.17±2.22 and 6.86±1.90, respectively and these values for the six adenocarcinomas were 3.12±1.52, 3.80±1.94 and 4.88±1.49, respectively. The differences were not significant for the values of SUVmean, SUVmax and T/NT between the squamous cell carcinomas and adenocarcinomas (t=0.512, P=0.618; t=0.325, P=0.75; t=1.987, P=0.07; respectively).

Comparative analysis did not identify a statistical difference for the values of SUVmean, SUVmax and T/NT between groups 1 and 2 (P=0.603, 0.577 and 0.463, respectively). The SUVmean, SUVmax and T/NT values in group 1 were 4.01±2.14, 4.83±2.56 and 5.04±1.31, respectively and these values in group 2 were 3.46±1.58, 4.12±1.86 and 5.93±1.94, respectively (data not shown).

## Discussion

Several studies have assessed the value of ^11^C-Choline PET for detection and staging of lung cancer and the results have been controversial (15–17). The metabolism of choline in lung cancer cells has also been investigated in several studies (10–13,18–20). The majority of studies have suggested that phosphorylation is the only pathway of ^11^C-Choline metabolism in cancer cells (10–11), however, certain studies have demonstrated that acetylation is another pathway of choline metabolism in small cell lung cancer and acetylcholine acts as an autocrine growth factor in small cell lung cancer (12,13). In lung cancer cells, nicotinic acetylcholine receptor (nAChR) subunit transcripts have also been observed to be expressed at varying levels and one or more subunits are highly expressed in non-small cell lung carcinomas (18,19). In addition, the protein expression of nAChR subunits has been investigated (18,20). This suggests that the acetylation pathway is important in lung cancer.

The present study demonstrated that the mRNA and protein expression of ChAT and ChoK increased in nine and 14 of the 18 lung tumors, respectively, compared with the expression in normal lung tissues. A total of eight of the 18 cases exhibited a high expression of ChAT and ChoK. The only case with high expression of ChAT was identified to be mucoepidermoid carcinoma. The results indicated that the phosphorylation pathway was the main pathway of choline metabolism. There also exists an acetylation pathway in lung cancer and the acetylation pathway may exist not only in small cell lung cancer but also in non-small cell lung cancer. It is reasonable to consider that the increased mRNA and protein expression of ChAT was associated with the high expression of nAChR in lung cancer cells.

However, three out of the 18 tumors exhibited no expression of ChAT and ChoK, but exhibited a high uptake of ^11^C-Choline, which may be due to other factors, including high expression of nAChR, the oxidation pathway or tumor blood flow. In addition, the SUV and T/NT values between groups 1 and 2 did not demonstrate statistical significance, which was another representation of the choline metabolism in lung cancer; however why the case without expression of ChAT and ChoK exhibited a high uptake of ^11^C-Choline remains to be elucidated. These results require further investigation in future studies.

The present study has several limitations. Firstly, the number of patients who enrolled in the present study was limited. The small population may lead to biased results. Secondly, the present study included cases of lung cancer, without benign lesions. Certain studies have revealed that specific types of benign lesions, including inflammatory granulation tissue exhibit a high uptake of ^11^C-Choline (7,21). Identification of the ^11^C-Choline metabolic mechanism in benign lesions may assist in differentiating malignant and benign lesions. In addition, the mRNA and protein expression of ChAT and ChoK were investigated. Other factors, including nAChR expression, the oxidation pathway and tumor blood flow may also have a role in ^11^C-Choline uptake and metabolism, which are to be investigated in a future study.

In conclusion, all lung tumor lesions were visualized using ^11^C-Choline PET/CT imaging. The phosphorylation and acetylation pathways may be important in ^11^C-Choline metabolism in different pathological types of lung cancer.

## Figures and Tables

**Table I:** Primers used for reverse transcription polymerase chain reaction.

Gene	Primer	Length (bp)
*ChAT*	F: 5′-GGAGATGTTCTGCTGCTATG-3′ R: 3′-GGAGGTGAAACCTAGTGGCA-5′	280
*ChoK*	F: 5′-ATCCCACCAAGAAACAACAGC-3′ R: 5′-TGGTGGAAATAGGCATCAAAC-3′	260
*β-actin*	F: 5′-GTGGGGCGCCCAGGCACCAC-3′ R: 5′-CTCCTTAATGTCACGCACGATTT-3′	550

ChaT, choline acetyltransferase; ChoK choline kinase; F, forward; R, reverse.
